# Clinical and Molecular Characterization of Myeloid Sarcoma: A Systematic Review and Meta-Analysis

**DOI:** 10.3390/cancers17243975

**Published:** 2025-12-12

**Authors:** Dakshin Sitaram Padmanabhan, Jeff Justin Aguilar, Sushmitha Nanja Reddy, Asmita Shukla, Vikram Dhillon, Sikander Chohan, Anisha Rajavel, Razan Alhaddad, Ella Hu, Janaka S. S. Liyanage, Jay Yang, Suresh Kumar Balasubramanian

**Affiliations:** 1 Department of Hematology and Oncology, Karmanos Cancer Institute, Wayne State University, Detroit, MI 48201, USA; 2 Department of Hematology and Oncology, Neal Cancer Center/Houston Methodist Hospital, Houston, TX 77030, USA; 3 Department of Pathology, Wayne State University, Detroit, MI 48201, USA; 4 Shiffman Medical Library, Wayne State University, Detroit, MI 48201, USA; 5 Biostatistics and Bioinformatics Core, Department of Oncology, Wayne State University, Detroit, MI 48201, USA; 6 Department of Translational Hematology and Oncology Research, Taussig Cancer Institute, Cleveland Clinic Foundation, Cleveland, OH 44195, USA

**Keywords:** myeloid sarcoma, extramedullary acute myeloid leukemia, molecular profiling, systematic review, meta-analysis

## Abstract

**Background/Objectives**: Myeloid sarcoma (MS) is a rare extramedullary manifestation of myeloid blasts, with limited systematic data, particularly regarding molecular (NGS) concordance between MS tissue and bone marrow. We hypothesized that clonal heterogeneity may exist between these sites due to their distinct biological environments. **Methods**: We conducted a systematic review and meta-analysis of 85 studies encompassing 7241 MS patients, to evaluate clinical characteristics, mutational profiles, treatment patterns, and outcomes. Mutational concordance or discordance between MS and bone marrow was assessed in a subset of 112 patients. **Results**: Male predominance (59%) and skin/soft tissue localization (31%) were most common. *NPM1* (25%) and *FLT3* (20%) were the most frequently reported mutations. Among 112 patients with paired sequencing, 56% showed discordance in mutational profiles. *NPM1* was significantly enriched in MS sites compared to bone marrow (35% vs. 21%, *p* = 0.02) and was associated with skin involvement. Discordance was more frequent in isolated and secondary MS. Venetoclax with hypomethylating agents achieved a 44% response rate, mainly in secondary MS. Post-transplant isolated extramedullary relapse occurred in 46% of relapsed patients and was linked to high rates of graft-versus-host disease. The pooled median overall survival was 12.8 months. **Conclusions**: MS demonstrates significant molecular heterogeneity. Routine site-specific NGS profiling may guide targeted therapy in this rare disease.

## Simple Summary

Myeloid sarcoma (MS) is biologically and clinically distinct from acute myeloid leukemia. Despite the use of AML-directed therapies, long-term outcomes in MS remain poor and generally inferior to many AML subtypes. Systematic studies of MS reporting clinical and treatment data are limited, with fewer studies reporting data on molecular concordance or discordance in paired NGS of the MS site and bone marrow. Paired NGS of bone marrow and the MS site is not routinely performed in clinical practice. Using a meta-analytical approach to aggregate data from existing studies, we found that *NPM1* mutations are enriched in MS sites compared to bone marrow with a high rate of molecular discordance, in addition to high rates of GVHD in post-HSCT relapse as MS and modest efficacy to VEN + HMA combination regimens. These findings support the prioritization of NGS sequencing in the MS site so that mutations can be targeted appropriately.

## 1. Introduction

Myeloid sarcoma (MS) is biologically and clinically distinct from acute myeloid leukemia (AML), partly driven by aberrant molecular interactions that facilitate tissue-homing and retention [1]. MS may present concomitantly with marrow-involved AML or as an isolated lesion without detectable bone marrow disease [2,3,4].

The overall incidence of MS is about 2.5–9% of patients with synchronous AML, and is commonly associated with the FAB M4 and M5 AML subtypes with monocytic morphology [5,6]. Isolated MS is less common (two cases per million adults), or <1% of AML cases at presentation [5]. MS can present in the pediatric population as well as in adults, though commonly between the fourth and sixth decades of life [4]. There is a slight male predominance [7], and MS can occur in any tissue, but the most commonly reported sites include the skin (leukemia cutis) and soft tissue, followed by lymph nodes, the gastrointestinal tract, bone, testis, peritoneum, and central nervous system [5]. Like AML, MS sequencing studies have revealed somatic mutations, notably in *NPM1*, followed by *FLT3* and RTK-RAS pathway mutations. Inversion 16, t(8;21), 11q23, t(8;17), monosomy 7, and chromosome 5q deletion are among the common chromosomal abnormalities observed in MS [8]. Pediatric myeloid sarcoma has a distinct mutational profile, with a higher incidence of inv16, t(8;21), and *KMT2A* rearrangements, thus suggesting different biologic drivers in this subset of patients [9].

MS can be locally symptomatic due to the mass effect at a particular site/organ, or associated with cytopenia when there is concordant bone marrow infiltration, and might have systemic symptoms like anorexia and weight loss often related to AML disease progression in general [10]. Though tissue biopsy is key for confirming the diagnosis after a thorough physical examination, imaging modalities, particularly FDG PET/CT, can be used to distinguish between other pathologies, such as abscesses and hematomas, and also to determine the size, number, and location of lesions [11,12].

The pathogenetic mechanism in MS is incompletely understood, but is proposed to be due to the overexpression of adhesion molecules, such as neural cell adhesion molecule CD56 and the interaction of leukemic cell surface markers like lymphocyte function-associated antigen-1 (LFA-1) with intracellular adhesion molecule-1 (ICAM-1), which may promote extramedullary homing of blasts [1]. A high expression of the CXC chemokine ligand 4 (CXCR4) and the activation of the CXCR4-CXCL12 axis may be associated with homing to the extramedullary niche where the leukemia cells proliferate [13].

The relatively high incidence of MS post-allogeneic hematopoietic stem cell transplantation (allo-HSCT) further supports the hypothesis that MS possesses a pathogenetic mechanism that can escape the graft-versus-leukemia effect, as well as chemotherapy at sanctuary sites [14]. Despite the use of AML-directed induction regimens, often combined with localized radiotherapy, surgical resection, and transplantation, long-term outcomes in MS remain poor and generally inferior to those of many AML subtypes [4,15].

Studies examining the clinical and molecular effects of myeloid sarcoma are still limited, primarily because of a lack of more granular exploratory studies on MS than just regular pathological evaluation. Although evolving targeted therapies have shown promise in AML, there is no standardized approach to perform next-generation sequencing (NGS) on both bone marrow and extramedullary lesions simultaneously, since the differential enrichment of actionable myeloid mutations between marrow and MS may carry critical therapeutic implications. Clonal heterogeneity of somatic myeloid mutations between bone marrow and MS sites likely reflects distinct biological niches and may influence both disease behavior and response to targeted agents. Scant literature reports on molecular concordance or discordance in paired NGS analyses reflect both variability in clinical practice and conventional grouping of MS under the same broader AML treatment paradigm. This underscores the need for a comprehensive and systematic review and meta-analysis of published studies to elucidate the clinical and molecular landscape of MS. Large-scale aggregation of existing data may yield transformative insights that inform precision diagnostics and guide the application of novel therapies in this challenging condition.

In this study, we performed a meta-analysis of the largest cohort of MS to date, in addition to patient data from our experience, to primarily ascertain if discordance in NGS mutational profiles existed between the MS site and bone marrow. Demonstrating such molecular differences would emphasize the need to routinely perform paired NGS on both MS lesions and bone marrow, thereby guiding treatment decisions in the era of mutation-specific targeted therapies for this unique disease and enabling comprehensive management of AML. With the evolving treatment landscape in AML, we also sought to describe treatment trends of post-transplant MS relapse cases and the efficacy of venetoclax–hypomethylating agent (VEN + HMA) combination regimens in MS management.

## 2. Materials and Methods

### 2.1. Search Strategy

A PubMed, Web of Science, EMBASE, and Scopus literature search involving all available research articles pertaining to MS from 1999 to 2025 was conducted using keywords as Medical Subject Headings (MeSH) search terms. A summary of the search strategy is further given in Supplementary Data S1. Three researchers (D.P, J.A, and E.H) independently searched for articles published within this search query. Our systematic review and meta-analysis strictly followed the PRISMA (Preferred Reporting Items for Systematic Reviews and Meta-Analyses) guidelines, as mentioned in Supplementary Data S2.

### 2.2. Selection Criteria and Data Collection

Studies included in the meta-analysis were either retrospective or prospective cohort studies with results pertaining to our primary and secondary research objectives. Articles excluded were articles not in English, a purely pediatric population with patients <18 years of age, review articles, case reports, and case series of less than ten patients. Articles which met our inclusion criteria were initially scrutinized based on titles and abstracts and later evaluated in their entirety. The eligibility of studies was independently evaluated by two researchers (D.P, J.A). In the event of a dispute pertaining to the inclusion of a particular study, unanimity was gained through further review with two other investigators (S.K.B, V.D). Covidence systematic review software (www.covidence.org, date accessed on 9 October 2024) was used to manage and streamline the systematic review process [16].

The primary objective of the study was to study the prevalence of somatic NGS mutations in MS and molecular concordance/discordance between MS sites and the bone marrow. Molecular concordance was defined as the exact same mutational profile in both the MS site and the bone marrow samples in terms of NGS mutations detected; while discordance was any difference in the mutational profile between the MS site and bone marrow.

The secondary objectives included the study of clinical, demographic, and laboratory parameters, as well as treatment prevalence, analysis of post-transplant extramedullary relapse as MS, and the impact of VEN + HMA treatment regimens. To look at the prevalence of mutations across studies that included NGS mutation data, we included data of our clinical cohort at Karmanos Cancer Institute (KCI) of thirteen MS patients.

The study protocol was registered in The International Prospective Register of Systematic Reviews (PROSPERO) under two submissions, which encompass the primary (CRD42023450876) and secondary (CRD42023445255) objectives, respectively.

### 2.3. Statistical Analysis

Using R software (version 4.3.1), a meta-analysis was performed to calculate pooled prevalence estimates for the following proportional data: descriptive characteristics of MS patients, MS tumor site distribution, mutation frequencies, mutational concordance/discordance, and treatment data. The Freeman–Tukey double arcsine transformation was applied to stabilize variance, and the pooled estimates were calculated using an inverse variance random effects model [17]. Pooled estimates of median age, median laboratory parameters, and median overall survival were calculated using the methods of McGrath et al., which estimates the sample means and standard deviations from reported medians and interquartile ranges [18]. The assessment of publication bias was performed through the generation of funnel plots using the funnel function from the meta R package. Statistical testing of publication bias was performed using a mixed-effects model to test for asymmetry in the proportion meta-analysis [19]. To assess the concordance and discordance of gene mutations between bone marrow and MS tissue, 2 × 2 contingency tables were generated by pooling available data across studies. A subsequent meta-analysis was performed by using the rma.peto function to calculate the pooled odds ratios, comparing mutation frequencies between tissue types.

## 3. Results

**Figure 1:**
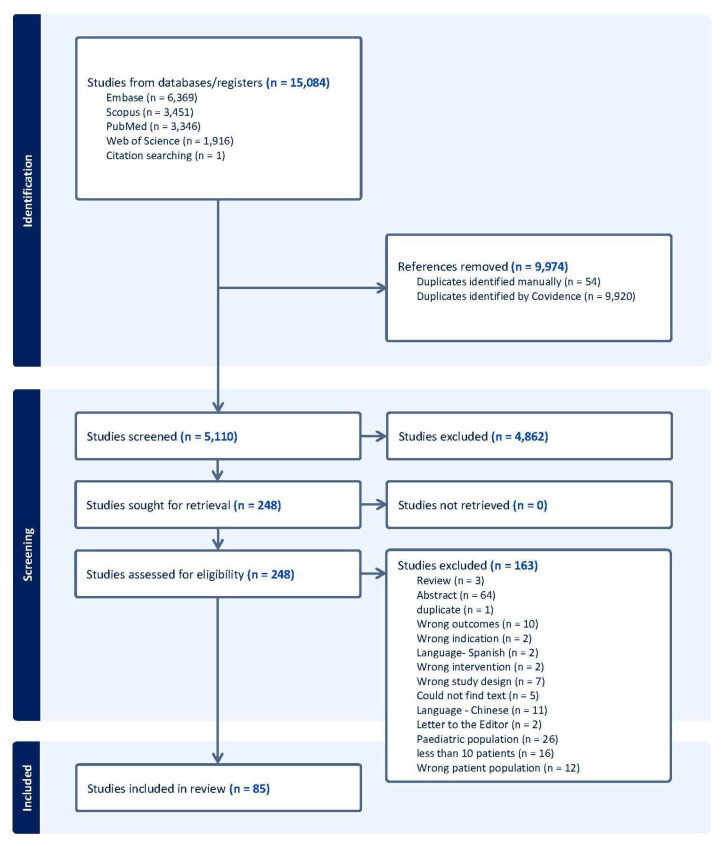
PRISMA Diagram depicting literature review and article selection process.

A total of 15,084 studies were obtained from PubMed, EMBASE, Scopus, and Web of Science using the search strategy previously mentioned, of which 9974 duplicate studies were removed. A total of 5110 studies were screened, and 4862 studies were excluded by the exclusion criteria. A total of 248 studies were assessed for further eligibility and full-text review. A total of 85 studies [2,14,15,20,21,22,23,24,25,26,27,28,29,30,31,32,33,34,35,36,37,38,39,40,41,42,43,44,45,46,47,48,49,50,51,52,53,54,55,56,57,58,59,60,61,62,63,64,65,66,67,68,69,70,71,72,73,74,75,76,77,78,79,80,81,82,83,84,85,86,87,88,89,90,91,92,93,94,95,96,97,98,99,100,101] were included in the final analysis, which included 7241 MS patients as the largest cohort studied in the literature to our knowledge (Figure 1).

### 3.1. Demographic and Clinical Parameters

Demographic and clinical parameters in MS showed a male gender preponderance (59% of patients), which was distributed similarly across all the studies (I^2^ = 29%, *p* = 0.01; Figure S1). The median age of patients across studies was 46 years (95% CI 46–53). Across studies, patients were found to have low median Hb at 10 g/dL, leukocytosis at 22 × 10^3^/ µL, and thrombocytopenia at 71 × 10^3^/µL.

**Figure 2:**
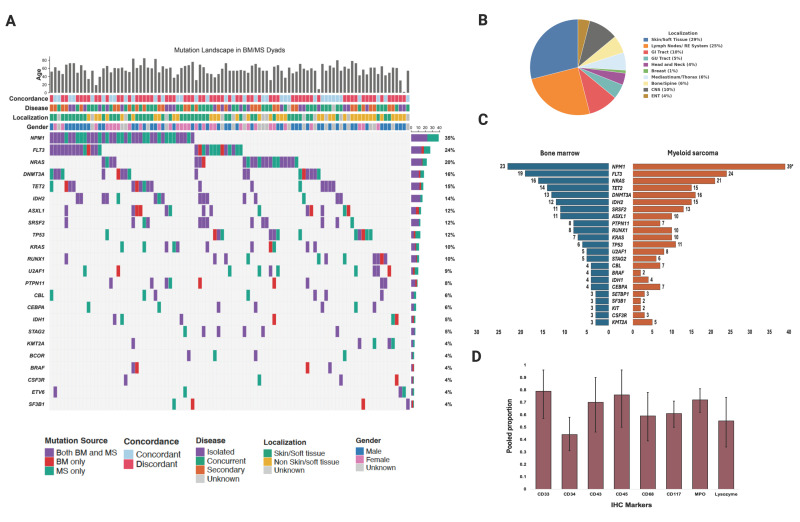
(**A**) Mutational landscape of bone marrow and myeloid sarcoma site dyads. (**B**) Localization of myeloid sarcoma. (**C**) Tornado plot showing patient frequencies of NGS mutations in paired MS site/bone marrow samples; * denotes *p* < 0.05. (**D**) Immunohistochemistry markers pooled prevalence in myeloid sarcoma.

The most common tissue localization of MS was the skin/soft tissue (29%), followed by the lymph nodes/reticuloendothelial system (25%), and gastrointestinal tract and central nervous system (10%) (Figure 2B). Meta-analysis across studies showed the prevalence of skin/soft tissue localization was 31% (95% CI 27–35%), but the distribution was heterogeneous across studies (I^2^ = 90%, *p* < 0.0001; Figure S2). Isolated MS was prevalent in 27% of patients, concurrent MS in 61%, and secondary/therapy-related MS in 28% of patients; however, the distribution was highly variable. Abnormal karyotype was seen in 53% of patients, with 52% falling in the ELN intermediate risk and 30% in the ELN high-risk groups. A total of 87% of patients received chemotherapy, 29% received radiotherapy, 16% surgery, and 41% received HSCT as part of their treatment regimen (Table 1) [2,15,20,21,22,24,25,26,27,28,29,30,31,32,33,34,35,36,37,38,39,40,41,42,43,45,47,48,49,51,52,54,56,57,58,59,60,61,62,64,65,66,67,68,69,70,72,73,74,75,76,77,78,79,80,81,83,86,87,88,89,90,91,92,93,94,95,96,97,99].

On comparison of studies with purely isolated MS (n = 9) versus purely concurrent MS (n = 13) cohorts, there was no difference in male gender distribution (57% vs. 56%, *p* = 0.86). However, there was a significant difference with respect to localization to the skin and soft tissue in isolated MS versus concurrent MS (40% vs. 24%, *p* < 0.0001). There was also a notably increased number of patients who received allo-HSCT in concurrent MS compared to isolated MS (69% vs. 12%, *p* < 0.0001).

In terms of immunohistochemical (IHC) markers that were found in the MS tissue biopsy samples from studies which reported this data, the IHC markers with the highest pooled prevalence were CD33 (79%), CD45 (76%), and MPO (72%) (Figure 2D). CD56 had the least pooled prevalence on IHC (27%).

### 3.2. Molecular Characterization

Paired bone marrow/MS site DNA sequencing data (n = 112, including 4 patients from KCI) revealed discordance in mutational profile between the MS site and bone marrow in 63 (56%) patients and concordance in the rest [25,29,31,41,49,58,78,81,94,101]. *NPM1* was the most frequent mutation in the MS sample (39 patients) and enriched in the MS site compared to the bone marrow (35% vs. 21%, *p* = 0.02) (Figure 2A,C). *FLT3* was the next most common mutation observed in the MS site, but there was only a numerical enrichment in MS compared to the bone marrow (22% vs. 17%, *p* = 0.72). *TP53* mutations were more prevalent in the MS site compared to bone marrow; however, the difference was not significant (10% vs. 5%, *p* = 0.20). There was no significant difference in the prevalence of concordance/discordance in relation to localization to skin/soft tissue versus non-skin/soft tissue sites. *NPM1* mutant MS compared to wild-type (WT) patients were more likely to localize to the skin/soft tissue (74% vs. 45%, *p* = 0.007). Patients with discordant mutations were more likely to have isolated MS or secondary MS compared to concurrent MS (72% vs. 75% vs. 35%, *p* = 0.0003).

A meta-analysis of concordance/discordance patterns between the MS site and bone marrow to assess odds ratio (OR) was also performed to determine particular enrichment, but no statistically significant OR was obtained (Table S1). Notably, *NPM1* mutations were around three times more likely to be present in the MS site compared to the bone marrow on meta-analysis; however, the difference was not statistically significant [OR 2.80 (95% CI 1.32–5.95); *p* = 0.43].

**Figure 3:**
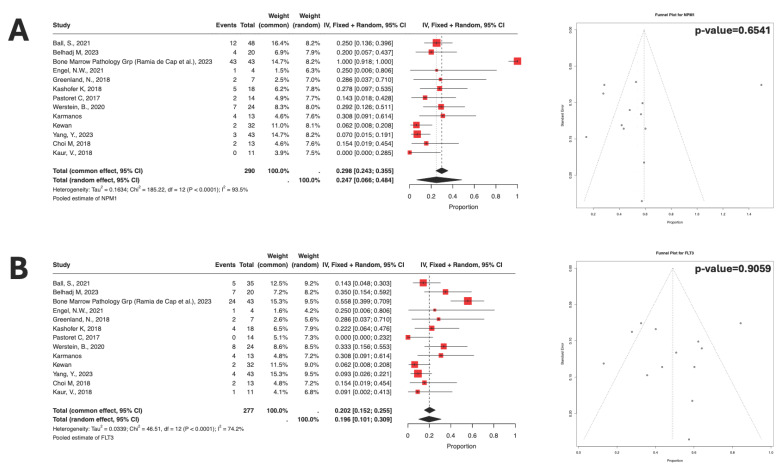
(**A**) Forest plot of *NPM1*. (**B**) *FLT3* prevalence with funnel plots for publication bias testing; both from studies (in order): [29,31,41,49,58,78,81,94], current study, [15,37,59,96].

Mutational analysis in the bone marrow samples across studies showed *NPM1*^MT^ to be more prevalent at 25%, followed by *FLT3*^MT^ at 20% (Figure 3A,B) and *NRAS*^MT^ at 12% (Table 1). Again, as noted previously, there was significant heterogeneity in the distribution of the prevalence of these mutations across studies.

### 3.3. Treatment Data

Studies which specifically described VEN + HMA combination regimens for the treatment of MS showed that most patients were males (60%) and 44% of patients had skin or soft tissue MS localization. The majority had secondary/therapy-related MS (70%) and 52% of patients had isolated MS. A third of patients (29%) presented with a normal karyotype and a third (32%) had an intermediate ELN risk classification, while the remaining (31%) were classified as ELN high-risk. With respect to treatment option with the type of HMA used, 34% had decitabine (DEC) and 66% had azacitidine (AZA) used in the regimen. A total of 37% of patients were treated with radiotherapy, 6% had local surgery, and 38% had prior HSCT as part of their treatment. CR/CRi was noted in 44% of patients, while a partial response was seen in 8% of patients and no response in 46% of patients (Table 2; Figure S3) [56,62,76,100].

Among patients with extramedullary relapse of acute leukemia as MS post-HSCT, isolated extramedullary relapse was noted in 46% of patients, while extramedullary and bone marrow relapse (EMR + BMR) was seen in 16% of patients. Acute GVHD occurred in 29% of patients, while chronic GVHD was seen in 32% of patients (Table 3; Figure S4) [14,23,44,46,50,53,55,63,71,82,84,85,98].

**Figure 4:**
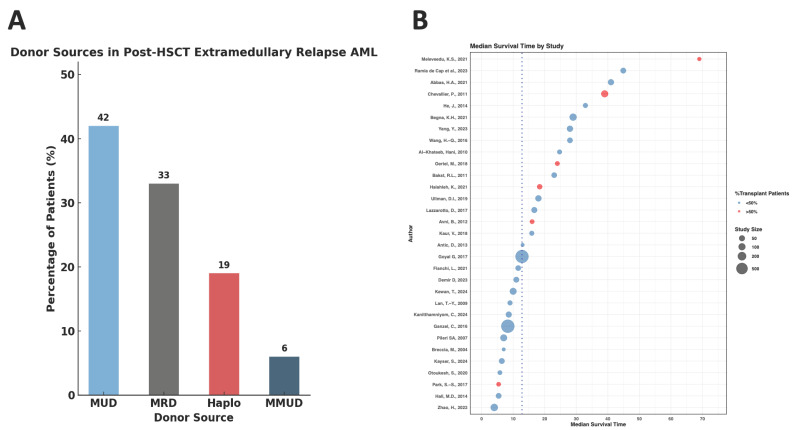
(**A**) Donor sources in post-HSCT extramedullary relapse AML. (**B**) Median survival time by study, stratifying by proportion of HSCT patients- from studies (in order): [2,15,20,21,26,27,29,30,34,36,39,42,45,47,51,52,54,57,59,62,64,65,69,75,76,77,81,91,92,96,99].

From the available data on patients with MS post-transplant, 42% had a matched unrelated donor (MUD) transplant (Figure 4A). On meta-median analysis of the studies, the median overall survival (OS) was analyzed to be 12.8 months. Studies that reported higher OS tended to have a proportion of HSCT as part of the treatment in >50% of patients (Figure 4B).

### 3.4. Case Series from Our Experience

**Figure 5:**
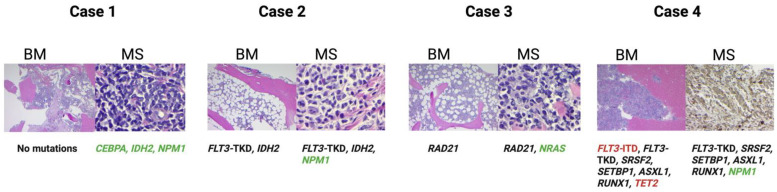
Differential mutational profiles of the bone marrow and myeloid sarcoma from the Karmanos experience. Cases 1, 2, and 3 show histopathology H&E slides from the bone marrow and corresponding myeloid sarcoma specimens. Case 4 shows the H&E slide of the bone marrow, while the myeloid sarcoma specimen highlights CD68 positive cells.

Four patients treated at our center showed distinct mutational profiles on NGS of the bone marrow and MS that were clearly discordant. Case 1, a 50-year-old female with relapsed MS about five years post-HSCT for AML, presented with a right breast mass with *CEBPA* (VAF 11%), *IDH2* (VAF 40%), and *NPM1* (VAF 39%) mutations while bone marrow at that time did not show any mutations. Case 2, a 68-year-old male treated with 7 + 3 induction chemotherapy followed by HiDAC consolidation and midostaurin for *FLT3*-mutated AML, presented with relapsed MS in the form of a skin rash, most pronounced on the right anterior shoulder. Bone marrow NGS showed mutations in *FLT3*-TKD (VAF 1%) and *IDH2* (VAF 1%), while MS mutations were *FLT3*-TKD (VAF 46%), *IDH2* (VAF 43%), and *NPM1* (VAF 34%). Case 3, a 50-year-old male treated with 7 + 3 induction chemotherapy followed by HiDAC consolidation and allo-HSCT, presented with a right cheek swelling nearly ten years post-HSCT. Bone marrow NGS revealed *RAD21* mutation (51%), while the MS right cheek sample showed *NRAS* (VAF 43%) and *RAD21* (VAF 50%) mutations. Case 4, a 69-year-old male with secondary AML from prior MDS, presented with facial numbness and multiple subcutaneous nodules in the chest and axilla and was started on VEN + AZA induction. BM NGS showed *FLT3*-ITD mutation (VAF non-estimable), *FLT3*-TKD (VAF 37%), *ASXL1* (VAF 44%), *RUNX1* (VAF 55%), *SRSF2* (VAF 58%), *SETBP1* (VAF 48%) and *TET2* (24%); MS NGS from the left chest nodule reported mutations in *ASXL1* (VAF 44%), *FLT3*-TKD (VAF 66%), *RUNX1* (VAF 33%), *SETBP1* (VAF 39%), *SRSF2* (VAF 44%), and *NPM1* (VAF 27%). This patient’s sarcomatous lesions improved remarkably once gilteritinib was added to the 2nd cycle of VEN +AZA; however, menin inhibitors were not available at that time for the targeting *NPM1* mutation. The differential mutational profiles between bone marrow and MS are summarized in Figure 5.

## 4. Discussion

In our largest meta-analysis of MS cohorts to date, we demonstrate demographic trends, molecular patterns, and treatment outcomes in this less explored disease. A male preponderance among the MS patients homogeneously seen across all studies in our meta-analysis was strikingly similar to AML demographics, which also has the same gender predilection, in contrast to the median age of presentation for MS being younger (59 years) than the historically reported 68 years for AML [102]. This demographic difference further incites in-depth associational research into genes associated with sex chromosomes and predilection to MS. The significant link between isolated MS and skin involvement compared to concurrent MS could be from the skin’s conducive environment for extramedullary myelopoiesis, the tissue-homing mechanisms of leukemic cells, and the absence of marrow disease that probably allows the skin to serve as a sanctuary site [103]. In concurrent MS, systemic disease with bone marrow involvement may contribute to the spread of the disease to other organs, making the skin less likely to be the exclusive site of involvement.

Sequencing studies between the MS and bone marrow paired samples significantly validated the prevalence of molecular discordance in more than half of the patients; however, this was in contrast to previous reports where molecular concordance was reported to be more common (70%) [104]. Importantly, *NPM1* and *FLT3* were the most common mutations in the MS samples, with *NPM1* being more enriched in the MS sample compared to the bone marrow. These findings are significant, especially in the era of targeted therapies, with menin inhibitors being brought to the frontline treatment of *NPM1*-mutated AML [105], which could in turn be used in treating MS, only if the molecular architecture of MS and AML is clarified in all patients. The overall pooled prevalence of *NPM1* mutations in MS from our meta-analysis (25%) was higher than the historically reported prevalence of 15% from a prior study [106].

It is also noted from our results that MS patients with *NPM1* mutations had lesions localized to the skin and soft tissue compared to non-skin/soft tissue sites. As reported earlier, *NPM1* mutations are frequently associated with leukemia cutis with an OR of 3 [107]. Perhaps the most important observation by a study comparing the survival outcomes of *NPM1*-mutated MS vs. NPM1-mutated AML was that OS was significantly shorter for *NPM1*-mutated MS (45 mos vs. 93 mos [81]. Given that survival outcomes are poor in this subset of MS compared to AML, with an enrichment clearly noted in MS sites compared to the bone marrow and with a predilection to the skin, NGS sequencing of both the MS and bone marrow may be clinically informative for treatment decisions. It is also important to explore these survival numbers again with the advent of menin inhibition in *NPM1* mutant AML.

*TP53* mutations were exclusively present in some MS patients at the MS site and not in the bone marrow, and though not statistically significant, primarily due to lower sample size, they may define another subset of MS patients associated with a poor prognosis, similar to what is observed in *TP53*-mutated AML [108]. The lack of a significant difference between MS and bone marrow samples for *FLT3* mutations suggests that, while *FLT3* may drive leukemic transformation in the marrow, its role in MS might be more in conjunction with the primary site and less site-dependent. Except for two studies that reported discordance, all others showed molecular concordance with respect to *FLT3* mutations [25,101]. Despite the absence of OS data in most molecular studies, it was observed in one study that patients with mutational discordance between the bone marrow and MS site had worse OS than patients with concordance, and specifically had targetable NGS mutations such as *NPM1* and *IDH2* enriched in the MS site [94].

Another observation from our study was that patients with discordance in mutations between the MS and the bone marrow samples were more likely to have isolated or secondary/therapy-related MS compared to concurrent MS, underscoring a possible mechanistic explanation in concurrent MS. The bone marrow and extramedullary tumor are typically seeded by the same leukemic clone simultaneously, while isolated MS may have an independent clonal evolution and secondary/therapy-related MS may have a divergent clonal branching under therapy-related stress.

VEN + HMA combination regimens are presently the standard of care in elderly/unfit AML patients [109]. In MS, it revealed a notable efficacy (CR/CRi rate of 44%), and most patients (70%) had secondary MS. In one study, the CR rate was significantly better in patients with newly diagnosed MS, at 64% [62]. More studies exploring this combination regimen in MS are needed to draw conclusions regarding its efficacy in both newly diagnosed MS and secondary MS. The poor outcomes observed in secondary/therapy-related MS in this group suggest that prior treatment may have modulated the tumor microenvironment, further complicating therapeutic strategies.

Epigenetic dysregulation appears to play a critical role in the biology of MS and may aid in explaining some of the clonal differences we observed between MS lesions and bone marrow samples. Prior genomic and transcriptomic studies have shown that MS tissue often carries distinct DNA methylation patterns and chromatin-modifying signatures compared with paired bone marrow samples, suggesting that the extramedullary microenvironment imposes unique selective pressures that shape epigenetic remodeling [49,96]. Aberrant promoter hypermethylation and alterations in chromatin regulators have also been described and may contribute to immune evasion, homing behavior, and persistence in sanctuary sites [4,6]. Mutations affecting epigenetic regulators—including *DNMT3A*, *TET2*, *IDH1/2*, *ASXL1*, and *KMT2A* rearrangements—are not uncommon in MS and further support the contribution of epigenetic mechanisms to its pathogenesis [7,15,58]. Of note, *NPM1*-mutated MS, which we found to be enriched at MS sites, is closely linked to menin-*KMT2A*-mediated transcriptional dysregulation, providing a clear therapeutic rationale for the use of menin inhibitors in this subset [81,110]. The modest but reproducible activity of VEN + HMA regimens in MS across published cohorts also suggests that epigenetic targeting strategies remains clinically relevant in this disease [76,109]. Collectively, these findings indicate that MS is shaped not only by genetic alterations but also by site-specific epigenetic evolution—reinforcing the need for paired marrow/MS sequencing and driver-directed therapies in future studies.

Extramedullary relapse as MS post-allo-HSCT in AML patients is frequently reported, with rates as high as 5–12% [111]. Among the 13 studies that examined the prevalence of isolated EMR and BMR, we noted a higher prevalence of isolated EMR as compared to EMR + BMR (46% vs. 16%). One study showed improved survival rates in isolated EMRs compared with EMR + BMR (27.5% vs. 11.1%, *p* < 0.05) [98]. Furthermore, we noticed relatively high rates of acute and chronic GVHD in 29% and 32% of patients, which may be explained by a reduced graft-versus-leukemia effect in an EMR vs. BMR [112,113].

It should be noted that two prior meta-analyses have examined related aspects of MS and extramedullary disease (EMD), although each addressed different questions from those explored in the present study. Untaaveesup et al. evaluated 37 studies encompassing 5646 patients across pediatric and adult populations to characterize the prevalence of genetic alterations in MS, identifying *FLT3*-ITD as the most common mutation (17.5%) and RUNX1::RUNX1T1 as the most frequent fusion (28.5%), with *NPM1* detected in 17% of MS patients [114]. In contrast, our pooled estimates demonstrated a higher prevalence of *NPM1* (25%) and a lower relative prevalence of *FLT3* (20%). Their analysis did not assess molecular concordance or discordance between MS and paired bone marrow samples, which represents a central theme of our work. Lin et al. examined the prognostic impact of EMD in AML across 13 studies, reporting increased mortality among patients with EMD (HR 1.49) and suggesting that HSCT mitigates this adverse effect; however, their review focused broadly on EMD-related survival outcomes and did not explore MS-specific molecular heterogeneity, features of MS relapse post-HSCT, or outcomes associated with contemporary therapies such as VEN + HMA [115]. By addressing these biologic and clinical dimensions—particularly concordance/discordance patterns and detailed post-transplant and treatment-specific characteristics—our study provides complementary and novel insights that extend the existing literature.

While this extensive meta-analysis of MS, a rare diagnostic entity in itself, provides some valuable insights, some limitations should be addressed. Molecular concordance and discordance patterns between the MS site and the bone marrow were explored in very few studies in the literature, and hence, the sample size while conducting its analysis is limited, leading to inadequate statistical power to demonstrate statistical differences. Next, inherent to any meta-analysis using published data, publication bias is likely to have been introduced as studies tend to report positive or unique findings rather than negative or equivocal ones. Thirdly, while analyzing clinical parameters and therapeutic options in MS, there were notably high heterogeneity (I^2^) values across studies, which could potentially undermine the reliability of combined pooled prevalence of the parameters studied. Fourth, the impact of allo-HSCT in terms of OS in MS, while intriguing, could not be definitively assessed in our analysis owing to significant heterogeneity in the type of treatment received across studies and inconsistent reporting of survival durations. Lastly, head-to-head comparisons of isolated and concurrent MS clinical characteristics and MS versus AML clinical differences, which were certainly other enticing areas of interest, could not be clarified owing to the same limitation with respect to the heterogeneous reporting of clinical parameters.

## 5. Conclusions

In conclusion, this study emphasizes the importance of exploring molecular concordance/discordance patterns between the MS site and bone marrow as an important clinical tool to guide treatment options in the age of targeted therapies. *NPM1* mutations were enriched in MS sites compared to the bone marrow, a critical piece of information that might potentially have a positive impact, especially with the advent of menin inhibition and other mutation-targeting agents. Extramedullary relapse of AML post allo-HSCT as MS is an important clinical phenomenon which should not be overlooked, and is associated with high rates of acute and chronic GVHD. VEN + HMA combination therapies in MS, particularly in secondary MS, show modest efficacy and should be explored further.

## Figures and Tables

**Table 1:** Meta-analysis of clinical characteristics of myeloid sarcoma.

Parameter		Studies (n)	Patients (n)	Events (n)	Proportion (%, 95% CI)	Heterogeneity, I^2^ (%)	*p*-Value
Diagnosis	Isolated MS	70	6945	2219	27 (15–40)	99	****
	Concurrent MS	70	6945	4408	61(47–75)	99	****
	Secondary MS	40	3403	703	28 (19–37)	96	***
Abnormal Karyotype		37	1411	719	53 (45–61)	86	***
ELN Risk	Favorable	15	1230	163	14 (10–19)	68	***
	Intermediate	15	1230	741	52 (44–60)	81	***
	High-Risk	15	1230	326	30 (23–38)	79	***
Treatment	Chemotherapy	43	3495	2610	87(80–93)	96	***
	Radiotherapy	32	2510	599	29 (21–38)	95	***
	Surgery	21	1855	368	16 (9–25)	94	***
	HSCT	45	4720	2367	41 (24–60)	99	***
Mutation prevalence	*DNMT3A* ^MT^	13	289	41	10 (5–17)	53	*
	*TET2* ^MT^	13	290	36	11 (7–15)	0	ns
	*ASXL1* ^MT^	13	290	28	7 (3–12)	28	ns
	*NPM1* ^MT^	13	290	87	25 (7–48)	94	***
	*FLT3* ^MT^	13	277	64	20 (10–31)	74	***
	*NRAS* ^MT^	13	289	42	12 (6–19)	56	**
	*IDH1* ^MT^	13	290	17	4 (1–7)	0	ns
	*IDH2* ^MT^	13	290	27	7(4–11)	0	ns
	*TP53* ^MT^	13	289	23	6 (2–11)	39	ns
	*RUNX1* ^MT^	13	289	19	4 (2–8)	15	ns

Based on random effects model; ns—not significant, * *p* < 0.05, ** *p* < 0.01, *** *p* < 0.0001, **** *p* < 1 × 10^−6^.

**Table 2:** Meta-analysis of venetoclax–hypomethylating combination therapy in myeloid sarcoma.

Parameter		Studies (n)	Patients (n)	Events (n)	Proportion (95% CI) *	Heterogeneity, I^2^ (%)	*p*-Value
Male Gender		4	89	53	60 (49–70)	0	ns
Skin/Soft Tissue Localization		4	89	32	44 (20–68)	78	**
Diagnosis	Isolated	4	89	37	52 (23–80)	85	**
	Secondary	4	89	57	70 (46–90)	77	**
Normal Karyotype		4	82	24	29 (12–48)	63	***
ELN Risk	Favorable	4	85	14	16 (5–31)	53	ns
	Intermediate	4	85	29	32 (22–43)	0	ns
	High-Risk	4	85	42	31 (21–42)	67	**
Treatment	VEN + DEC	4	89	30	38 (3–82)	94	***
	VEN + AZA	4	89	52	40 (4–85)	94	***
	Radiotherapy	3	43	15	37 (10–69)	76	*
	Surgery	4	89	6	6 (0–22)	70	*
	Prior HSCT	4	89	30	38 (17–61)	75	**
Response	CR/CRi	4	89	39	44 (33–55)	0	ns
	PR	4	89	9	8 (1–19)	43	ns
	No Response	4	89	41	46 (31–61)	44	ns

Based on random effects model; ns–not significant, * *p* < 0.05, ** *p* < 0.01, *** *p* < 0.0001.

**Table 3:** Meta-analysis of extramedullary relapse of AML as MS post-HSCT characteristics.

Parameter	Studies (n)	Patients (n)	Events (n)	Proportion (95% CI) *	Heterogeneity, I^2^ (%)	*p*-Value
Isolated EMR	11	326	130	46 (25–67)	93	***
EMR + BMR	11	326	60	16 (6–29)	86	***
Acute GVHD	11	327	86	29 (16–44)	87	***
Chronic GVHD	11	327	99	32 (18–48)	88	***

Based on random effects model; * *p* < 0.05, *** *p* < 0.001.

## Data Availability

Data generated or analyzed during this study are included in Supplementary Materials. Further information regarding datasets generated during the current study are available from the corresponding author on reasonable request.

## Supplementary Materials

The following supporting information can be downloaded at https://www.mdpi.com/article/10.3390/cancers17243975/s1, Figure S1: Meta-Analysis of Male Gender Across Myeloid Sarcoma Studies; Figure S2: Meta-Analysis of Skin/Soft Tissue Localization Across Myeloid Sarcoma Studies; Figure S3: Meta-Analysis of Treatment Response in MS Patients Treated with Venetoclax–Hypomethylating Agent Combination Regimens; Figure S4: Meta-Analysis of Acute and Chronic GvHD Incidence in Extramedullary AML relapse as MS post-transplant; Table S1: Meta-Analysis of Concordance/Discordance Mutation Patterns in Myeloid Sarcoma/Bone Marrow Dyads. Supplementary Data S1 contains the database search strategy for myeloid sarcoma studies. Supplementary Data S2 shows the PRISMA 2020 checklist for our systematic review and meta-analysis. Reference [116] is cited in the supplementary materials.

## Abbreviations

The following abbreviations are used in this manuscript:

MS	Myeloid sarcoma
HSCT	Hematopoietic stem cell transplant
GVHD	Graft-versus-host disease
CR	Complete response
