# Supplementary material for: Clinical and Molecular Characterization of Myeloid Sarcoma: A Systematic Review and Meta-Analysis
Source: Cancers (Basel). 2025 Dec 12;17(24):3975. doi: 10.3390/cancers17243975 (PMC12730967; doi:10.3390/cancers17243975)
Supplement: Supplementary file 1 [file cancers-17-03975-s001.zip › cancers-3973514-supplementary.pdf]

Supplementary Appendix to ***Clinical and Molecular Characterization of Myeloid Sarcoma: A Systematic Review and Meta-Analysis***

Figure S1: Meta-Analysis of Male Gender Across Myeloid Sarcoma Studies

Study	Events	Total	Weight (common)	Weight (random)	IV, Fixed + Random, 95% CI
Abbas, H.A., 2021	36	56	0.9%	1.5%	0.643 [0.504; 0.766]
Al-Khateeb, Hani, 2010	14	21	0.4%	0.7%	0.667 [0.430; 0.854]
Alexiev, B.A., 2007	6	13	0.2%	0.4%	0.462 [0.192; 0.749]
Anekpuritanang T, 2021	17	38	0.6%	1.1%	0.447 [0.286; 0.617]
Ansari-Lari, M.A., 2004	13	20	0.3%	0.6%	0.650 [0.408; 0.846]
Antic, D., 2013	8	12	0.2%	0.4%	0.667 [0.349; 0.901]
Avni, B., 2012	13	19	0.3%	0.6%	0.684 [0.434; 0.874]
Bakst, R.L., 2011	25	38	0.6%	1.1%	0.658 [0.486; 0.804]
Ball, S., 2021	25	45	0.7%	1.3%	0.556 [0.400; 0.704]
Begna, K.H., 2021	70	109	1.8%	2.4%	0.642 [0.545; 0.732]
Belhadj M, 2023	31	46	0.8%	1.3%	0.674 [0.520; 0.805]
Bianchi, S., 2022	.	13	0.0%	0.0%	
Ramia de Cap et al., 2023	22	43	0.7%	1.2%	0.512 [0.355; 0.667]
Bourlon, C., 2017	24	39	0.6%	1.1%	0.615 [0.446; 0.766]
Breccia, M., 2004	8	12	0.2%	0.4%	0.667 [0.349; 0.901]
Chen, WY., 2013	10	20	0.3%	0.6%	0.500 [0.272; 0.728]
Chevallier, P., 2011	57	99	1.6%	2.3%	0.576 [0.472; 0.675]
Choi M, 2018	30	62	1.0%	1.6%	0.484 [0.355; 0.614]
Claerhout, H., 2018	35	72	1.2%	1.8%	0.486 [0.367; 0.607]
Demir D, 2023	27	45	0.7%	1.3%	0.600 [0.443; 0.743]
Eckardt, J.-N., 2022	119	225	3.7%	3.7%	0.529 [0.461; 0.596]
Engel, N.W., 2021	4	4	0.1%	0.1%	1.000 [0.398; 1.000]
Fianchi, L., 2021	22	38	0.6%	1.1%	0.579 [0.408; 0.737]
Fraccaroli, A., 2023	8	26	0.4%	0.8%	0.308 [0.143; 0.518]
Ganzel, C., 2016	445	769	12.6%	5.7%	0.579 [0.543; 0.614]
Goyal G, 2017	419	746	12.2%	5.7%	0.562 [0.525; 0.598]
Goyal, S.D., 2015	458	814	13.3%	5.8%	0.563 [0.528; 0.597]
Greenland, N., 2018	4	7	0.1%	0.2%	0.571 [0.184; 0.901]
Halahleh, K., 2021	22	32	0.5%	0.9%	0.688 [0.500; 0.839]
Hall, M.D., 2014	26	41	0.7%	1.2%	0.634 [0.469; 0.779]
He, J., 2014	8	23	0.4%	0.7%	0.348 [0.164; 0.573]
Jian, X., 2024	7	14	0.2%	0.5%	0.500 [0.230; 0.770]
Kanitthamniyom, C., 2024	25	53	0.9%	1.4%	0.472 [0.333; 0.614]
Kashofer K, 2018	13	18	0.3%	0.6%	0.722 [0.465; 0.903]
Kaur, V., 2018	16	23	0.4%	0.7%	0.696 [0.471; 0.868]
Kawamoto, K., 2016	97	131	2.2%	2.8%	0.740 [0.657; 0.813]
Kaygusuz, G., 2015	16	20	0.3%	0.6%	0.800 [0.563; 0.943]
Kayser, S., 2024	30	46	0.8%	1.3%	0.652 [0.498; 0.786]
Kewan, T., 2024	60	91	1.5%	2.2%	0.659 [0.553; 0.755]
Lan, T.-Y., 2009	14	24	0.4%	0.7%	0.583 [0.366; 0.779]
Lazzarotto, D., 2017	26	48	0.8%	1.3%	0.542 [0.392; 0.686]
Liang, J., 2023	280	472	7.7%	5.0%	0.593 [0.547; 0.638]
Lizardo-Thiebaud Maria, J., 2024	7	13	0.2%	0.4%	0.538 [0.251; 0.808]
Lontos, K., 2021	97	151	2.5%	3.0%	0.642 [0.560; 0.719]
Meleveedu, K.S., 2021	6	13	0.2%	0.4%	0.462 [0.192; 0.749]
Meyer, H.-J., 2019	75	151	2.5%	3.0%	0.497 [0.414; 0.579]
Movassaghian, M., 2015	190	345	5.7%	4.5%	0.551 [0.497; 0.604]
Mundada, M.C., 2024	13	24	0.4%	0.7%	0.542 [0.328; 0.744]
Nagamine, M., 2020	7	11	0.2%	0.4%	0.636 [0.308; 0.891]
Oertel, M., 2018	.	20	0.0%	0.0%	
Otoukesh, S., 2020	10	18	0.3%	0.6%	0.556 [0.308; 0.785]
Park, S.-S., 2017	12	17	0.3%	0.5%	0.706 [0.440; 0.897]
Pastoret C, 2017	8	14	0.2%	0.5%	0.571 [0.289; 0.823]
Patra, S., 2021	29	38	0.6%	1.1%	0.763 [0.598; 0.886]
Paydas, S., 2006	16	32	0.5%	0.9%	0.500 [0.319; 0.681]
Pileri SA, 2007	54	92	1.5%	2.2%	0.587 [0.479; 0.689]
Shan, M., 2021	43	74	1.2%	1.9%	0.581 [0.461; 0.695]
Shimizu, H., 2012	27	44	0.7%	1.2%	0.614 [0.455; 0.756]
Singhal, R.L., 2015	9	14	0.2%	0.5%	0.643 [0.351; 0.872]
Song, J.H., 2011	11	24	0.4%	0.7%	0.458 [0.256; 0.672]
Tsimberidou, A.-M., 2008	.	23	0.0%	0.0%	
Ullman, D.I., 2019	41	63	1.0%	1.6%	0.651 [0.520; 0.767]
Wang, H.-Q., 2016	20	39	0.6%	1.1%	0.513 [0.348; 0.676]
Wang, X., 2023	32	51	0.8%	1.4%	0.627 [0.481; 0.759]
Werstein, B., 2020	15	24	0.4%	0.7%	0.625 [0.406; 0.812]
Yamasaki, S., 2020	.	841	0.0%	0.0%	
Yang, Y., 2023	36	61	1.0%	1.6%	0.590 [0.457; 0.714]
Yu WJ, 2021	10	14	0.2%	0.5%	0.714 [0.419; 0.916]
Zhao, H., 2022	73	118	1.9%	2.6%	0.619 [0.525; 0.706]
Sun, J., 2024	93	162	2.7%	3.1%	0.574 [0.494; 0.651]
**Total (common effect, 95% CI)**		**6978**	**100.0%**	**.**	**0.582 [0.569; 0.595]**
**Total (random effect, 95% CI)**		**.**		**100.0%**	**0.588 [0.570; 0.606]**

Heterogeneity: $Tau^2 = 0.0011$; $Chi^2 = 91.22$, df = 65 ($P = 0.0177$); $I^2 = 28.7\%$

Pooled Estimate of Males

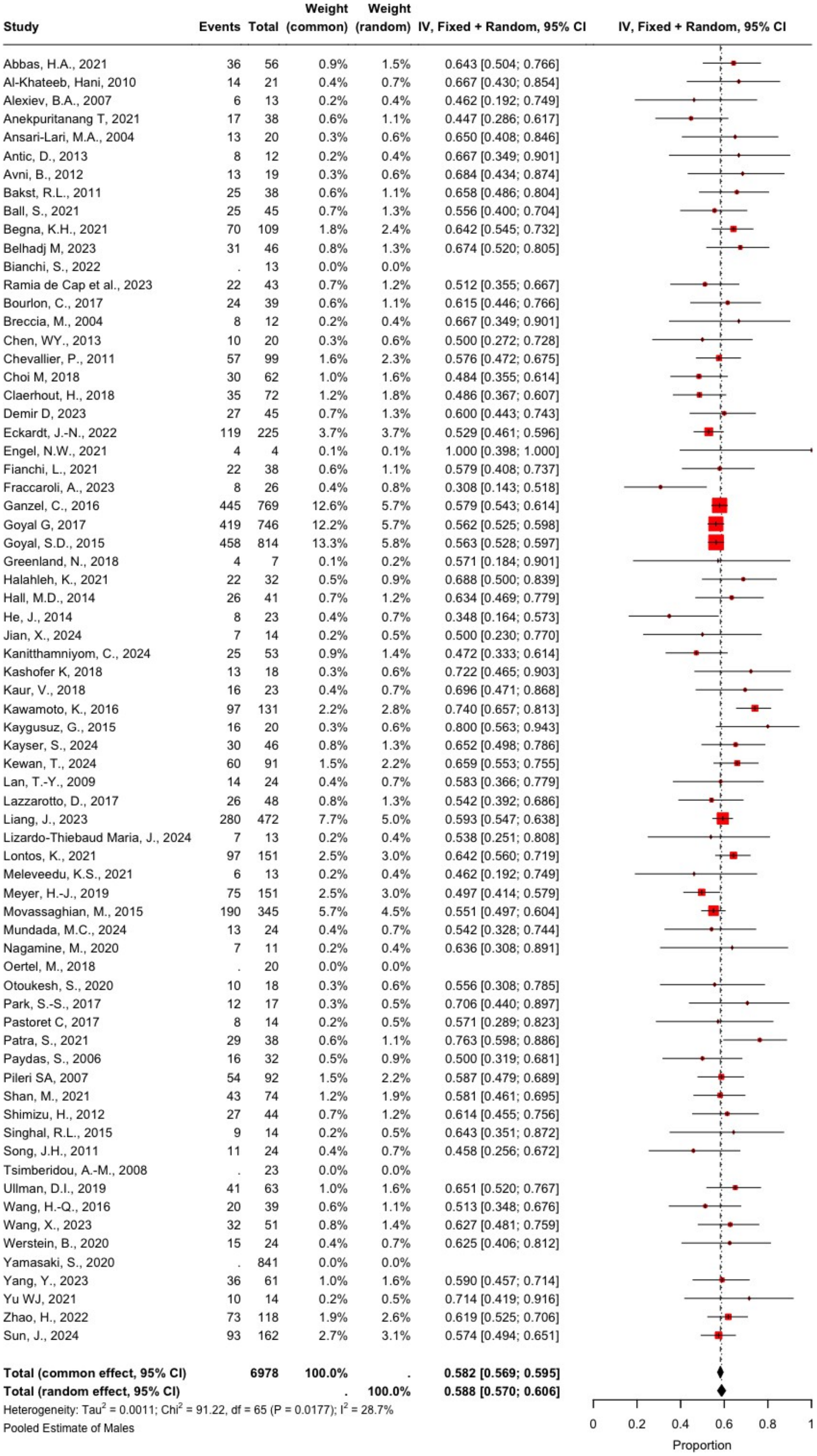

Figure S2: Meta-Analysis of Skin/Soft Tissue Localization Across Myeloid Sarcoma Studies

Study	Events	Total	Weight (common)	Weight (random)	IV, Fixed + Random, 95% CI
Abbas, H.A., 2021	19	56	0.9%	1.7%	0.339 [0.218; 0.478]
Al-Khateeb, Hani, 2010	4	21	0.3%	1.3%	0.190 [0.054; 0.419]
Alexiev, B.A., 2007	1	13	0.2%	1.1%	0.077 [0.002; 0.360]
Anekpuritanang T, 2021	9	38	0.6%	1.6%	0.237 [0.114; 0.402]
Ansari-Lari, M.A., 2004	8	20	0.3%	1.3%	0.400 [0.191; 0.639]
Antic, D., 2013	0	12	0.2%	1.1%	0.000 [0.000; 0.265]
Avni, B., 2012	6	19	0.3%	1.3%	0.316 [0.126; 0.566]
Bakst, R.L., 2011	0	38	0.6%	1.6%	0.000 [0.000; 0.093]
Ball, S., 2021	21	58	0.9%	1.7%	0.362 [0.240; 0.499]
Begna, K.H., 2021	37	109	1.7%	1.8%	0.339 [0.251; 0.436]
Belhadj M, 2023	23	46	0.7%	1.6%	0.500 [0.349; 0.651]
Ramia de Cap et al., 2023	25	43	0.7%	1.6%	0.581 [0.421; 0.730]
Bourlon, C., 2017	5	39	0.6%	1.6%	0.128 [0.043; 0.274]
Breccia, M., 2004	2	12	0.2%	1.1%	0.167 [0.021; 0.484]
Chen, WY., 2013	7	20	0.3%	1.3%	0.350 [0.154; 0.592]
Chevallier, P., 2011	24	63	1.0%	1.7%	0.381 [0.261; 0.512]
Choi M, 2018	2	13	0.2%	1.1%	0.154 [0.019; 0.454]
Claerhout, H., 2018	26	72	1.1%	1.7%	0.361 [0.251; 0.483]
Demir D, 2023	11	45	0.7%	1.6%	0.244 [0.129; 0.395]
Eckardt, J.-N., 2022	17	38	0.6%	1.6%	0.447 [0.286; 0.617]
Engel, N.W., 2021	2	4	0.1%	0.6%	0.500 [0.068; 0.932]
Fianchi, L., 2021	22	38	0.6%	1.6%	0.579 [0.408; 0.737]
Fraccaroli, A., 2023	12	26	0.4%	1.4%	0.462 [0.266; 0.666]
Ganzel, C., 2016	146	769	11.8%	1.9%	0.190 [0.163; 0.219]
Goyal G, 2017	326	746	11.5%	1.9%	0.437 [0.401; 0.473]
Goyal, S.D., 2015	155	814	12.5%	1.9%	0.190 [0.164; 0.219]
Greenland, N., 2018	3	7	0.1%	0.9%	0.429 [0.099; 0.816]
Halahleh, K., 2021	5	32	0.5%	1.5%	0.156 [0.053; 0.328]
Hall, M.D., 2014	16	41	0.6%	1.6%	0.390 [0.242; 0.555]
He, J., 2014	5	23	0.4%	1.4%	0.217 [0.075; 0.437]
Jian, X., 2024	6	14	0.2%	1.2%	0.429 [0.177; 0.711]
Kanitthamniyom, C., 2024	53	53	0.8%	1.7%	1.000 [0.933; 1.000]
Kashofer K, 2018	5	18	0.3%	1.3%	0.278 [0.097; 0.535]
Kaur, V., 2018	16	23	0.4%	1.4%	0.696 [0.471; 0.868]
Kawamoto, K., 2016	29	131	2.0%	1.8%	0.221 [0.154; 0.302]
Kaygusuz, G., 2015	5	20	0.3%	1.3%	0.250 [0.087; 0.491]
Kayser, S., 2024	9	46	0.7%	1.6%	0.196 [0.094; 0.339]
Kewan, T., 2024	49	91	1.4%	1.8%	0.538 [0.431; 0.644]
Lan, T.-Y., 2009	9	24	0.4%	1.4%	0.375 [0.188; 0.594]
Lazzarotto, D., 2017	23	48	0.7%	1.6%	0.479 [0.333; 0.628]
Liang, J., 2023	81	472	7.3%	1.9%	0.172 [0.139; 0.209]
Lizardo-Thiebaud Maria, J., 2024	6	13	0.2%	1.1%	0.462 [0.192; 0.749]
Lontos, K., 2021	58	151	2.3%	1.8%	0.384 [0.306; 0.467]
Meleveedu, K.S., 2021	4	13	0.2%	1.1%	0.308 [0.091; 0.614]
Meyer, H.-J., 2019	30	151	2.3%	1.8%	0.199 [0.138; 0.271]
Movassaghian, M., 2015	125	345	5.3%	1.9%	0.362 [0.312; 0.416]
Mundada, M.C., 2024	2	24	0.4%	1.4%	0.083 [0.010; 0.270]
Nagamine, M., 2020	3	11	0.2%	1.1%	0.273 [0.060; 0.610]
Oertel, M., 2018	4	20	0.3%	1.3%	0.200 [0.057; 0.437]
Otoukesh, S., 2020	9	18	0.3%	1.3%	0.500 [0.260; 0.740]
Park, S.-S., 2017	0	17	0.3%	1.3%	0.000 [0.000; 0.195]
Pastoret C, 2017	2	14	0.2%	1.2%	0.143 [0.018; 0.428]
Patra, S., 2021	5	38	0.6%	1.6%	0.132 [0.044; 0.281]
Paydas, S., 2006	8	32	0.5%	1.5%	0.250 [0.115; 0.434]
Pileri SA, 2007	26	92	1.4%	1.8%	0.283 [0.194; 0.386]
Shimizu, H., 2012	6	44	0.7%	1.6%	0.136 [0.052; 0.274]
Singhal, R.L., 2015	9	14	0.2%	1.2%	0.643 [0.351; 0.872]
Song, J.H., 2011	16	24	0.4%	1.4%	0.667 [0.447; 0.844]
Tsimberidou, A.-M., 2008	10	23	0.4%	1.4%	0.435 [0.232; 0.655]
Ullman, D.I., 2019	30	63	1.0%	1.7%	0.476 [0.349; 0.606]
Wang, H.-Q., 2016	4	39	0.6%	1.6%	0.103 [0.029; 0.242]
Wang, X., 2023	5	51	0.8%	1.6%	0.098 [0.033; 0.214]
Werstein, B., 2020	16	24	0.4%	1.4%	0.667 [0.447; 0.844]
Yamasaki, S., 2020	226	841	12.9%	1.9%	0.269 [0.239; 0.300]
Yang, Y., 2023	15	61	0.9%	1.7%	0.246 [0.145; 0.373]
Yu WJ, 2021	6	14	0.2%	1.2%	0.429 [0.177; 0.711]
Zhao, H., 2022	27	118	1.8%	1.8%	0.229 [0.157; 0.315]
**Total (common effect, 95% CI)**		**6470**	**100.0%**	.	**0.277 [0.265; 0.288]**
**Total (random effect, 95% CI)**			.	**100.0%**	**0.310 [0.268; 0.353]**

Heterogeneity: $Tau^2 = 0.0257$; $Chi^2 = 686.24$, $df = 66$ ($P < 0.0001$); $I^2 = 90.4\%$

Pooled Estimate of Skin & Soft Tissue

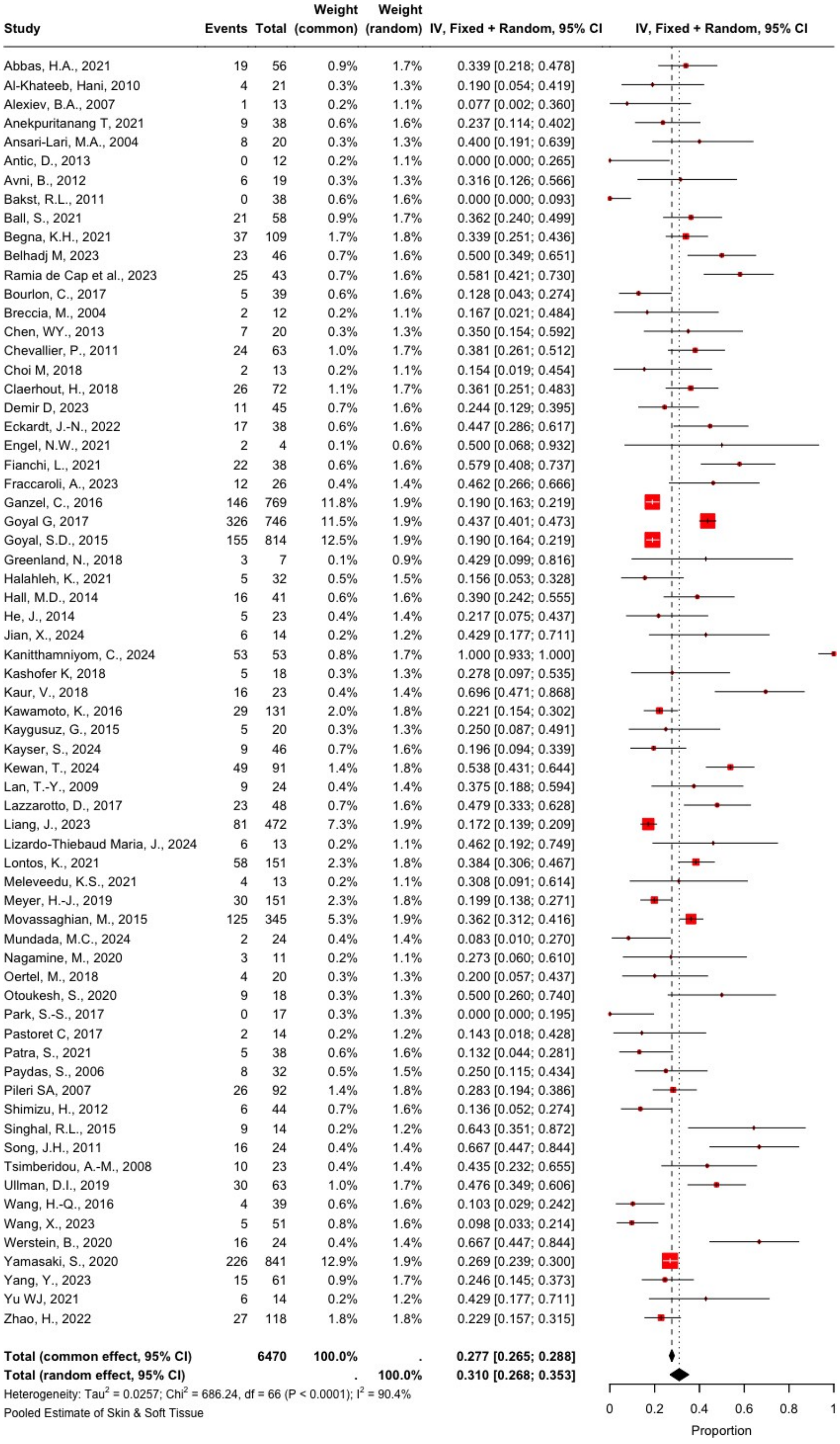

Figure S3: Meta-Analysis of Treatment Response in MS Patients Treated with Venetoclax-Hypomethylating Agent Combination Regimens

Study or Subgroup	Events	Total	Weight	IV, Random, 95% CI
**Response = CR/CRi**				
Bae S, 2024	3	11	7.3%	0.273 [0.060; 0.610]
Jian, X., 2024	8	14	7.9%	0.571 [0.289; 0.823]
Kayser, S., 2024	20	46	9.8%	0.435 [0.289; 0.589]
Otoukesh, S., 2020	8	18	8.4%	0.444 [0.215; 0.692]
**Total (95% CI)**		**89**	**33.3%**	**0.437 [0.331; 0.545]**
Heterogeneity: $Tau^2 = 0$; $Chi^2 = 2.06$, df = 3 (P = 0.5600); $I^2 = 0\%$				
**Response = PR**				
Bae S, 2024	1	11	7.3%	0.091 [0.002; 0.413]
Jian, X., 2024	3	14	7.9%	0.214 [0.047; 0.508]
Kayser, S., 2024	5	46	9.8%	0.109 [0.036; 0.236]
Otoukesh, S., 2020	0	18	8.4%	0.000 [0.000; 0.185]
**Total (95% CI)**		**89**	**33.3%**	**0.081 [0.011; 0.189]**
Heterogeneity: $Tau^2 = 0.0093$; $Chi^2 = 5.23$, df = 3 (P = 0.1556); $I^2 = 42.7\%$				
**Response = Relapsed/Refractory post-HMA/Ven**				
Bae S, 2024	7	11	7.3%	0.636 [0.308; 0.891]
Jian, X., 2024	3	14	7.9%	0.214 [0.047; 0.508]
Kayser, S., 2024	21	46	9.8%	0.457 [0.309; 0.610]
Otoukesh, S., 2020	10	18	8.4%	0.556 [0.308; 0.785]
**Total (95% CI)**		**89**	**33.3%**	**0.459 [0.308; 0.613]**
Heterogeneity: $Tau^2 = 0.0097$; $Chi^2 = 5.32$, df = 3 (P = 0.1496); $I^2 = 43.6\%$				
**Total (95% CI)**		**267**	**100.0%**	**0.308 [0.183; 0.447]**
Heterogeneity: $Tau^2 = 0.0436$; $Chi^2 = 53.16$, df = 11 (P < 0.0001); $I^2 = 79.3\%$				
Test for subgroup differences: $Chi^2 = 22.49$, df = 2 (P < 0.0001)				

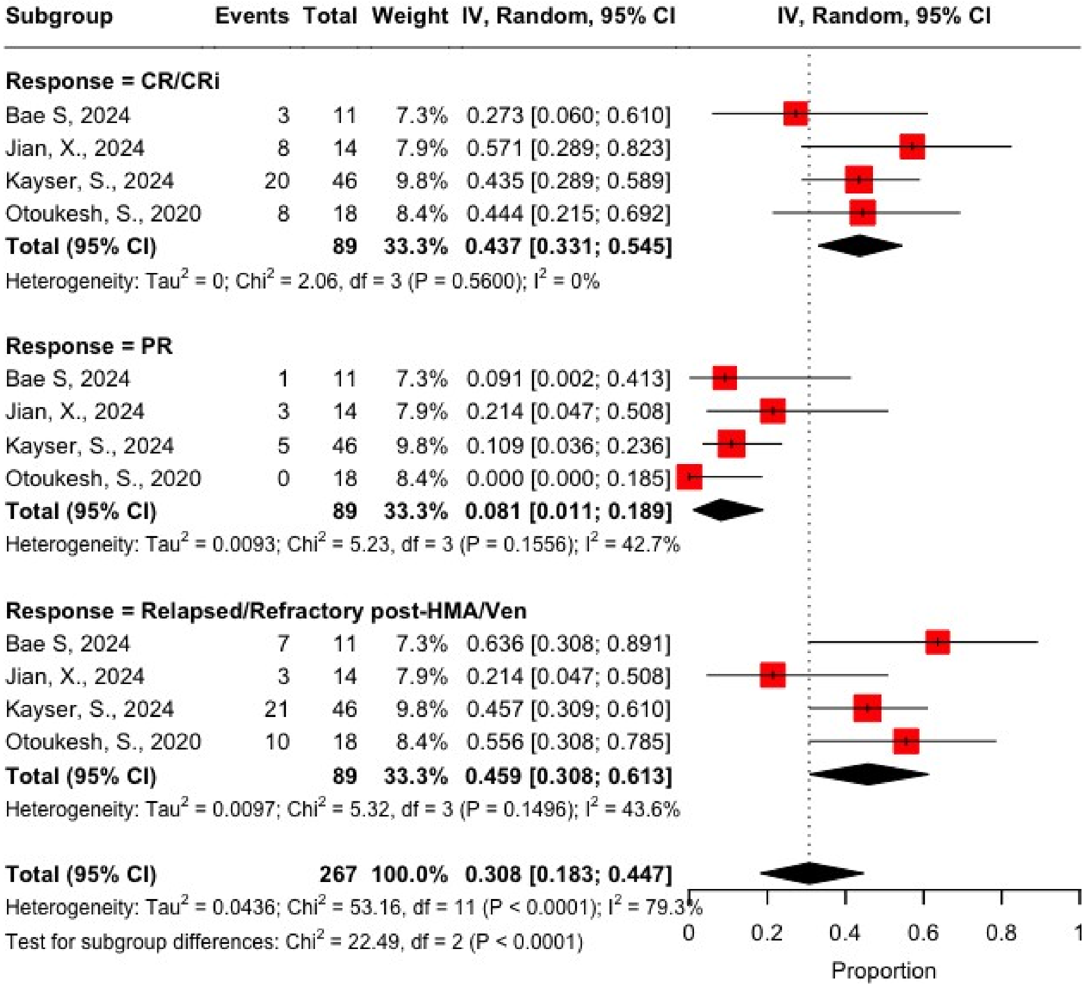

Figure S4: Meta-Analysis of Acute and Chronic GVHD Incidence in Extramedullary AML Relapse as MS post-transplant

Study	Events	Total	Weight (common)	Weight (random)	IV, Fixed + Random, 95% CI
Frietsch, J.J., 2021	8	19	5.9%	8.7%	0.421 [0.203; 0.665]
Ge, L., 2014	4	26	8.0%	9.1%	0.154 [0.044; 0.349]
Gunes, G., 2019	1	64	19.4%	10.0%	0.016 [0.000; 0.084]
Harris, A.C., 2013	15	26	8.0%	9.1%	0.577 [0.369; 0.766]
Huang, Q., 2014	11	28	8.6%	9.2%	0.393 [0.215; 0.594]
Kogut, N., 2013	10	18	5.6%	8.6%	0.556 [0.308; 0.785]
Mo, X.-D., 2014	7	40	12.2%	9.6%	0.175 [0.073; 0.328]
Sakellari, I, 2019	5	38	11.6%	9.6%	0.132 [0.044; 0.281]
Shem-Tov, N., 2017	8	31	9.5%	9.3%	0.258 [0.119; 0.446]
Shi, J.-M., 2013	4	12	3.8%	7.8%	0.333 [0.099; 0.651]
Solh, M., 2012	13	25	7.7%	9.1%	0.520 [0.313; 0.722]
**Total (common effect, 95% CI)**		**327**	**100.0%**	**.**	**0.231 [0.184; 0.280]**
**Total (random effect, 95% CI)**			**.**	**100.0%**	**0.292 [0.161; 0.441]**

Heterogeneity: $Tau^2 = 0.0544$; $Chi^2 = 74.48$, df = 10 ($P < 0.0001$); $I^2 = 86.6\%$

Pooled Estimate of Acute GvHD

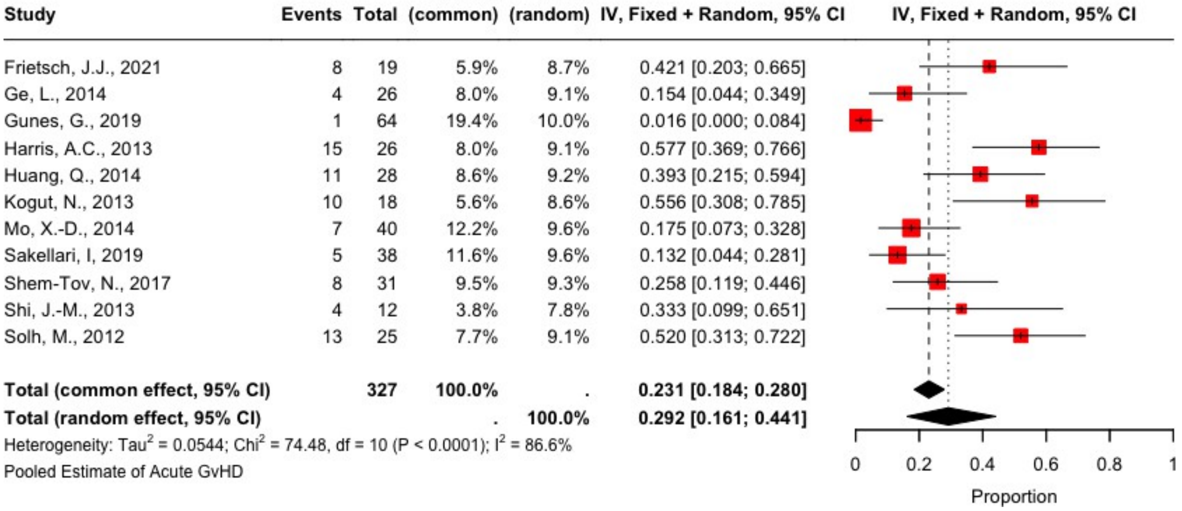

Study	Events	Total	Weight (common)	Weight (random)	IV, Fixed + Random, 95% CI
Frietsch, J.J., 2021	8	19	5.9%	8.7%	0.421 [0.203; 0.665]
Ge, L., 2014	1	26	8.0%	9.1%	0.038 [0.001; 0.196]
Gunes, G., 2019	5	64	19.4%	9.9%	0.078 [0.026; 0.173]
Harris, A.C., 2013	17	26	8.0%	9.1%	0.654 [0.443; 0.828]
Huang, Q., 2014	4	28	8.6%	9.2%	0.143 [0.040; 0.327]
Kogut, N., 2013	12	18	5.6%	8.6%	0.667 [0.410; 0.867]
Mo, X.-D., 2014	4	40	12.2%	9.5%	0.100 [0.028; 0.237]
Sakellari, I, 2019	18	38	11.6%	9.5%	0.474 [0.310; 0.642]
Shem-Tov, N., 2017	17	31	9.5%	9.3%	0.548 [0.360; 0.727]
Shi, J.-M., 2013	5	12	3.8%	8.0%	0.417 [0.152; 0.723]
Solh, M., 2012	8	25	7.7%	9.1%	0.320 [0.149; 0.535]
**Total (common effect, 95% CI)**		**327**	**100.0%**	**.**	**0.274 [0.225; 0.326]**
**Total (random effect, 95% CI)**			**.**	**100.0%**	**0.321 [0.177; 0.483]**

Heterogeneity: $Tau^2 = 0.0631$; $Chi^2 = 84.76$, df = 10 ($P < 0.0001$); $I^2 = 88.2\%$

Pooled Estimate of Chronic GvHD

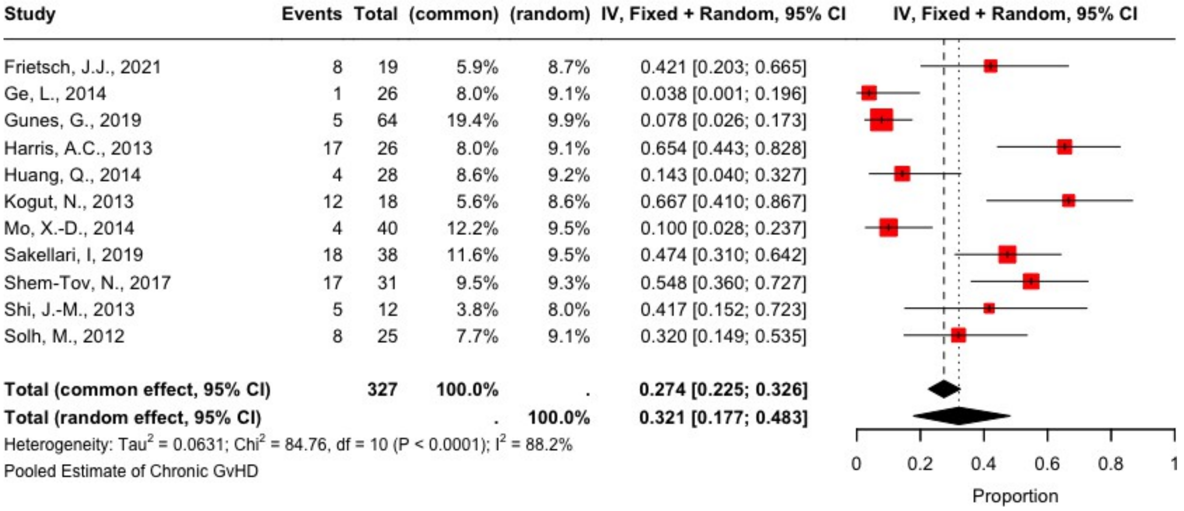

Table S1: Meta-Analysis of Concordance/Discordance Mutational Patterns in Myeloid Sarcoma/Bone Marrow Dyads

Parameter	Studies (n)	Patients (n)	BM^{MT} (n)	MS^{MT}(n)	Odds Ratio (95% CI)*
ASXL1^{MT}	5	49	11	9	0.78 (0.30-2.06)
DNMT3A^{MT}	5	76	8	10	1.29 (0.48-3.45)
FLT3^{MT}	6	73	13	15	1.21 (0.52-2.82)
IDH1^{MT}	3	57	4	2	0.50 (0.10-2.56)
IDH2^{MT}	7	87	11	14	1.33 (0.57-3.13)
NPM1^{MT}	7	86	20	34	**2.80 (1.32-5.95)**
RUNX1^{MT}	4	62	6	8	1.38 (0.45-4.17)
TP53^{MT}	5	70	6	10	1.76 (0.62-4.97)

***Based on fixed effects model**

Supplementary Data S1: Database Search Strategy for Myeloid Sarcoma Studies

PubMed

(((("isolated"[Title/Abstract] OR "de novo"[Title/Abstract]) OR (leukaemi*[Title/Abstract] OR leukemi*[Title/Abstract])) AND (("myeloid sarcoma"[Title/Abstract]) OR (sarcoma, myeloid[MeSH Terms]))) OR (("extramedullary"[Title/Abstract] OR "granulocytic sarcoma"[Title/Abstract] OR "chloroma"[Title/Abstract]) AND ("leukemia, myeloid, acute"[MeSH Terms] OR "AML"[Title/abstract] OR "acute myeloid leukemia"[Title/abstract] OR "acute myeloid leukaemia"[Title/Abstract]))) AND ((("human s"[All Fields] OR "humans"[MeSH Terms] OR "humans"[All Fields] OR "human"[All Fields]) NOT (("animals"[MeSH Terms:noexp] OR "animal"[All Fields]) NOT ("human s"[All Fields] OR "humans"[MeSH Terms] OR "humans"[All Fields] OR "human"[All Fields])))) AND (1999/01/01:2024/12/31[Date - Publication])

Embase

#17. #15 AND #16
#16. human NOT (animal NOT human)
#15. (#5 OR #13) AND [1999-2025]/py
#14. #5 OR #13
#13. #11 AND #12
#12. #9 OR #10
#11. #6 OR #7 OR #8
#10. 'acute myeloid leukemia':ti,ab OR 'acute myeloid leukaemia':ti,ab OR 'aml':ti,ab
#9. 'acute myeloid leukemia'/exp
#8. 'chloroma':ti,ab
#7. 'granulocytic sarcoma':ti,ab
#6. extramedullary:ti,ab
#5. #3 AND #4
#4. #1 OR #2
#3. 'granulocytic sarcoma'/exp
#2. leukemi*:ti,ab OR leukaemi*:ti,ab
#1. 'isolated':ti,ab OR 'de novo':ti,ab

Scopus

((TITLE-ABS-KEY (isolated OR "de novo" OR leukaemi* OR leukemi*) AND TITLE-ABS-KEY ("myeloid sarcoma"))) OR ((TITLE-ABS-KEY (extramedullary OR "granulocytic sarcoma" OR chloroma) AND TITLE-ABS-KEY (aml OR "acute myeloid leukemia" OR "acute myeloid leukaemia"))) AND PUBYEAR > 1998 AND PUBYEAR < 2026

Web of Science Core Collection

1: isolated OR “de novo” OR leukaemi* OR leukemi* (Topic) AND “myeloid sarcoma” (Topic)
2: extramedullary OR “granulocytic sarcoma” OR chloroma (Topic) AND AML OR “acute myeloid leukemia” OR “acute myeloid leukaemia" (Topic)
3: TS=(human or humans) NOT (TS=(animal or animals) NOT TS=(human or humans))
4: #1 OR #2
5: #4 AND PY=(1999-2025)

The study protocol was registered in The International Prospective Register of Systematic Reviews (PROSPERO) under two submissions which encompass the primary (CRD42023450876) and secondary (CRD42023445255) objectives respectively.

Supplementary Data S2: PRISMA 2020 Checklist [116]

Section and Topic	Item #	Checklist item	Reported, location/page
**TITLE**			
Title	1	Identify the report as a systematic review.	Yes, in title
**ABSTRACT**			
Abstract	2	See the PRISMA 2020 for Abstracts checklist.	Yes, abstract adheres to this
**INTRODUCTION**			
Rationale	3	Describe the rationale for the review in the context of existing knowledge.	Yes, pg.3
Objectives	4	Provide an explicit statement of the objective(s) or question(s) the review addresses.	Yes, pg.4 in Methods
**METHODS**			
Eligibility criteria	5	Specify the inclusion and exclusion criteria for the review and how studies were grouped for the syntheses.	Yes, pg.3
Information sources	6	Specify all databases, registers, websites, organisations, reference lists and other sources searched or consulted to identify studies. Specify the date when each source was last searched or consulted.	Yes, pg.3
Search strategy	7	Present the full search strategies for all databases, registers and websites, including any filters and limits used.	Yes, File S1
Selection process	8	Specify the methods used to decide whether a study met the inclusion criteria of the review, including how many reviewers screened each record and each report retrieved, whether they worked independently, and if applicable, details of automation tools used in the process.	Yes
Data collection process	9	Specify the methods used to collect data from reports, including how many reviewers collected data from each report, whether they worked independently, any processes for obtaining or confirming data from study investigators, and if applicable, details of automation tools used in the process.	Yes
Data items	10a	List and define all outcomes for which data were sought. Specify whether all results that were compatible with each outcome domain in each study were sought (e.g. for all measures, time points, analyses), and if not, the methods used to decide which results to collect.	Yes
	10b	List and define all other variables for which data were sought (e.g. participant and intervention characteristics, funding sources). Describe any assumptions made about any missing or unclear information.	Yes
Study risk of bias assessment	11	Specify the methods used to assess risk of bias in the included studies, including details of the tool(s) used, how many reviewers assessed each study and whether they worked independently, and if applicable, details of automation tools used in the process.	Yes, Covidence software was used
Effect measures	12	Specify for each outcome the effect measure(s) (e.g. risk ratio, mean difference) used in the synthesis or presentation of results.	Yes
Synthesis methods	13a	Describe the processes used to decide which studies were eligible for each synthesis (e.g. tabulating the study intervention characteristics and comparing against the planned groups for each synthesis (item #5)).	Yes
	13b	Describe any methods required to prepare the data for presentation or synthesis, such as handling of missing summary statistics, or data conversions.	Yes
	13c	Describe any methods used to tabulate or visually display results of individual studies and syntheses.	Yes, funnel plots using R
	13d	Describe any methods used to synthesize results and provide a rationale for the choice(s). If meta-analysis was performed, describe the model(s), method(s) to identify the presence and extent of statistical heterogeneity, and software package(s) used.	Yes
	13e	Describe any methods used to explore possible causes of heterogeneity among study results (e.g. subgroup analysis, meta-regression).	Yes
	13f	Describe any sensitivity analyses conducted to assess robustness of the synthesized results.	Yes
Reporting bias assessment	14	Describe any methods used to assess risk of bias due to missing results in a synthesis (arising from reporting biases).	Yes

PRISMA 2020 Checklist

Section and Topic	Item #	Checklist item	Reported, location/page
Certainty assessment	15	Describe any methods used to assess certainty (or confidence) in the body of evidence for an outcome.	Yes
**RESULTS**			
Study selection	16a	Describe the results of the search and selection process, from the number of records identified in the search to the number of studies included in the review, ideally using a flow diagram.	Yes
	16b	Cite studies that might appear to meet the inclusion criteria, but which were excluded, and explain why they were excluded.	Yes
Study characteristics	17	Cite each included study and present its characteristics.	Yes, all 85 studies have been cited
Risk of bias in studies	18	Present assessments of risk of bias for each included study.	Yes
Results of individual studies	19	For all outcomes, present, for each study: (a) summary statistics for each group (where appropriate) and (b) an effect estimate and its precision (e.g. confidence/credible interval), ideally using structured tables or plots.	Yes, Table 1, 2 and 3, Table S1, S2, S3
Results of syntheses	20a	For each synthesis, briefly summarise the characteristics and risk of bias among contributing studies.	Yes, example given for bias tested for mutations with funnel plot
	20b	Present results of all statistical syntheses conducted. If meta-analysis was done, present for each the summary estimate and its precision (e.g. confidence/credible interval) and measures of statistical heterogeneity. If comparing groups, describe the direction of the effect.	Yes
	20c	Present results of all investigations of possible causes of heterogeneity among study results.	Yes, in File S1
	20d	Present results of all sensitivity analyses conducted to assess the robustness of the synthesized results.	Yes
Reporting biases	21	Present assessments of risk of bias due to missing results (arising from reporting biases) for each synthesis assessed.	Yes
Certainty of evidence	22	Present assessments of certainty (or confidence) in the body of evidence for each outcome assessed.	Yes
**DISCUSSION**			
Discussion	23a	Provide a general interpretation of the results in the context of other evidence.	Yes
	23b	Discuss any limitations of the evidence included in the review.	Yes
	23c	Discuss any limitations of the review processes used.	Yes
	23d	Discuss implications of the results for practice, policy, and future research.	Yes
**OTHER INFORMATION**			
Registration and protocol	24a	Provide registration information for the review, including register name and registration number, or state that the review was not registered.	Yes
	24b	Indicate where the review protocol can be accessed, or state that a protocol was not prepared.	Yes
	24c	Describe and explain any amendments to information provided at registration or in the protocol.	Yes
Support	25	Describe sources of financial or non-financial support for the review, and the role of the funders or sponsors in the review.	Yes
Competing interests	26	Declare any competing interests of review authors.	Yes
Availability of data,	27	Report which of the following are publicly available and where they can be found: template data collection forms; data extracted from included studies;	Yes

PRISMA 2020 Checklist

Section and Topic	Item #	Checklist item	Reported, location/page
code and other materials		data used for all analyses; analytic code; any other materials used in the review.	

From: Page MJ, McKenzie JE, Bossuyt PM, Boutron I, Hoffmann TC, Mulrow CD, et al. The PRISMA 2020 statement: an updated guideline for reporting systematic reviews. BMJ 2021;372:n71. doi: 10.1136/bmj.n71. This work is licensed under CC BY 4.0. To view a copy of this license, visit https://creativecommons.org/licenses/by/4.0/
